# Recommendations for Using Health Service Coverage Cascades to Measure Effective Coverage for Maternal, Newborn, Child, and Adolescent Health Services or Interventions

**DOI:** 10.9745/GHSP-D-24-00158

**Published:** 2024-12-20

**Authors:** Kathleen Strong, Georgia Konstantinou, Ambrose Agweyu, Theresa Diaz, Debra Jackson, Minjoon Kim, Shogo Kubota, Hannah Leslie, Marzia Lazzerini, Tanya Marchant, Melinda Munos, Moise Muzigaba, Alicia Quach, Ashley Sheffel, Nuhu Yaqub, for the Life Stages Quality of Care Metrics Technical Working Group for Maternal, Newborn, Child and Adolescent Health and Ageing

**Affiliations:** a Department of Maternal, Newborn, Child and Adolescent Health and Aging, World Health Organization, Geneva, Switzerland.; b London School of Hygiene and Tropical Medicine, London, United Kingdom.; c KEMRI-Wellcome Trust Research Programme, Nairobi, Kenya.; d University of the Western Cape, Capetown, South Africa.; e Program Group, Health, UNICEF, New York, NY, USA.; f World Health Organization, Western Pacific Regional Office, Manila, Philippines.; g University of California, San Francisco, CA, USA.; h Institute for Maternal and Child Health IRCCS Burlo Garofolo, Trieste, Italy.; i Johns Hopkins Bloomberg School of Public Health, Baltimore, MD, USA.; j University of Melbourne, Melbourne, Australia.; k The World Bank Group, Global Financing Facility, Washington, DC, USA.

## Abstract

Using health service coverage cascades to measure effective coverage for maternal, newborn, child, and adolescent health services on a global scale is premature and requires further research and validation to reach consensus.

## Key Messages

- Measuring the contact coverage of maternal, newborn, child, and adolescent health (MNCAH) services overestimates the benefits of these services because the quality of care provided during a contact is not measured.
- Effective coverage measures the proportion of the population that received a service with sufficient quality to have a positive health outcome from the service.
- MNCAH services and interventions involve a series of complex interactions between patients and the health system that are best measured using health service coverage cascades that follow an individual through their care journey to identify where efficiency is lost.
- Current data limitations and gaps make measuring effective coverage using health service cascades challenging. Further investment in data collections and measurement methods is required so that the method can be used at both the facility and population levels.
- We argue that using these cascades for global monitoring for MNCAH is premature and make recommendations for how to scale up use of health service coverage cascades for global monitoring.

## FROM SIMPLE HEALTH SERVICE COVERAGE TO EFFECTIVE COVERAGE WITH QUALITY

As countries take on the challenge of meeting the Sustainable Development Goal targets for health (SDG 3)^1^ by 2030, they are monitoring a range of indicators, including those measuring coverage of essential health services for women, newborns, children, and adolescents. Existing coverage indicators measure service contact coverage or intervention coverage,^2^ that is, the proportion of the target population that either reaches an appropriate health service or receives the required intervention.

Within the context of this viewpoint, a health service is referred to as a system or organized effort aimed at providing health care to individuals or populations and that encompasses a wide range of components, including the infrastructure, personnel, resources, and processes necessary to deliver care (e.g., inpatient or outpatient services and emergency services).^3^ In contrast, a health intervention is a specific action or set of actions, such as a targeted approach to prevent, diagnose, or treat a particular health condition (e.g., administering a vaccine, performing a cesarean delivery, or a health education campaign), aimed at improving health outcomes.^4^ In other words, a health intervention is a specific measure taken within the broader context of a health service.

Service contact and intervention coverage indicators do not fully capture an important component of quality of care—the process of care, defined as what is actually done during the provision of such health services or intervention to help ensure the effectiveness, safety, people-centeredness, efficiency, timeliness, and equity of such processes. In fact, the current service contact coverage indicators only capture whether a woman or child has contact with a health service but not how these services are delivered and the degree to which they align with recommended practice. Intervention coverage indicators capture the proportion of the target population in need of the intervention that receives the intervention but do not fully capture the quality of intervention delivery. As a result, both intervention and service contact coverage indicators may overestimate the potential health benefits of an intervention or service.^5^^–^^8^

This observation, coupled with a slowing down in the reduction in annual rates of maternal, newborn, child, and adolescent (MNCA) mortality from preventable causes and a need to improve monitoring of SDG 3,^1^ has led to calls to move beyond intervention and service contact coverage measurements and toward measuring the effective coverage (EC) of a health service or intervention.^1^^,^^6^^–^^9^ EC aims to measure the proportion of the population in need of a health service or intervention that received a positive health outcome from a service or intervention.^6^^–^^8^ In this viewpoint, we discuss the challenges of measuring EC of maternal, newborn, child, and adolescent health (MNCAH) services and interventions in practice and make recommendations for how to improve EC measurement for MNCAH in the future.

We discuss the challenges of measuring EC of MNCAH services and interventions in practice and make recommendations for how to improve EC measurement for MNCAH in the future.

## APPROACHES TO MEASURING EFFECTIVE COVERAGE

One way to estimate EC is to use a set of carefully selected measures drawn from a health service cascade to calculate the expected health benefit that will accrue to the target population. Building on the work of Tanahashi and Amouzou et al., to further define EC indicators for MNCAH, the World Health Organization (WHO) and UNICEF convened the Effective Coverage Think Tank Group to make recommendations for standardizing the definition of EC and to decide on appropriate measurement approaches.^5^^–^^7^ The Think Tank Group recommended that EC be described using health service coverage cascades at the population level.^6^^,^^7^ Health service coverage cascades are a useful tool for evaluating health system performance by mapping the target population’s progress throughout their care journey and identifying areas where efficiency is lost. Each intervention is provided with a defined measure (i.e., the ratio between the number of people receiving the essential intervention and the target population for that service). A set of these measures represents the interaction between the service and the target population.

### Potential Use of Effective Coverage Measures at the Country Level

For MNCAH, essential interventions include a complex sequence of interactions between patients and the health system, allowing for an assessment of the health services along the MNCAH continuum of care.^6^^,^^10^ To address the challenges of capturing these interactions, the Think Tank Group adapted Amouzou et al.’s health service cascade, which summarizes a 7-step process moving from the target population (Step 1) through to the outcome-adjusted coverage (those having the expected positive outcome, Step 7) (Figure).^6^^,^^7^ The Think Tank group noted that the full cascade (i.e., measuring and reporting all relevant steps of the cascade for an intervention) is essential for monitoring at subnational and facility levels, where it can be used to identify bottlenecks in service delivery. The cascades can serve an important function for quality planning at the national level and for quality improvement at health facility or subnational levels, alerting program managers to areas where performance is suboptimal and aiding in the development of appropriate quality improvements to address the gaps. For example, a big drop between contact coverage and input-adjusted coverage in 1 or several health facilities may indicate that supplies and the equipment needed to provide the service are lacking in those settings. Action can be taken at the health facility or subnational level to improve facility infrastructure, with the intention of increasing the proportion of the target population that receives the service.

**FIGURE:**
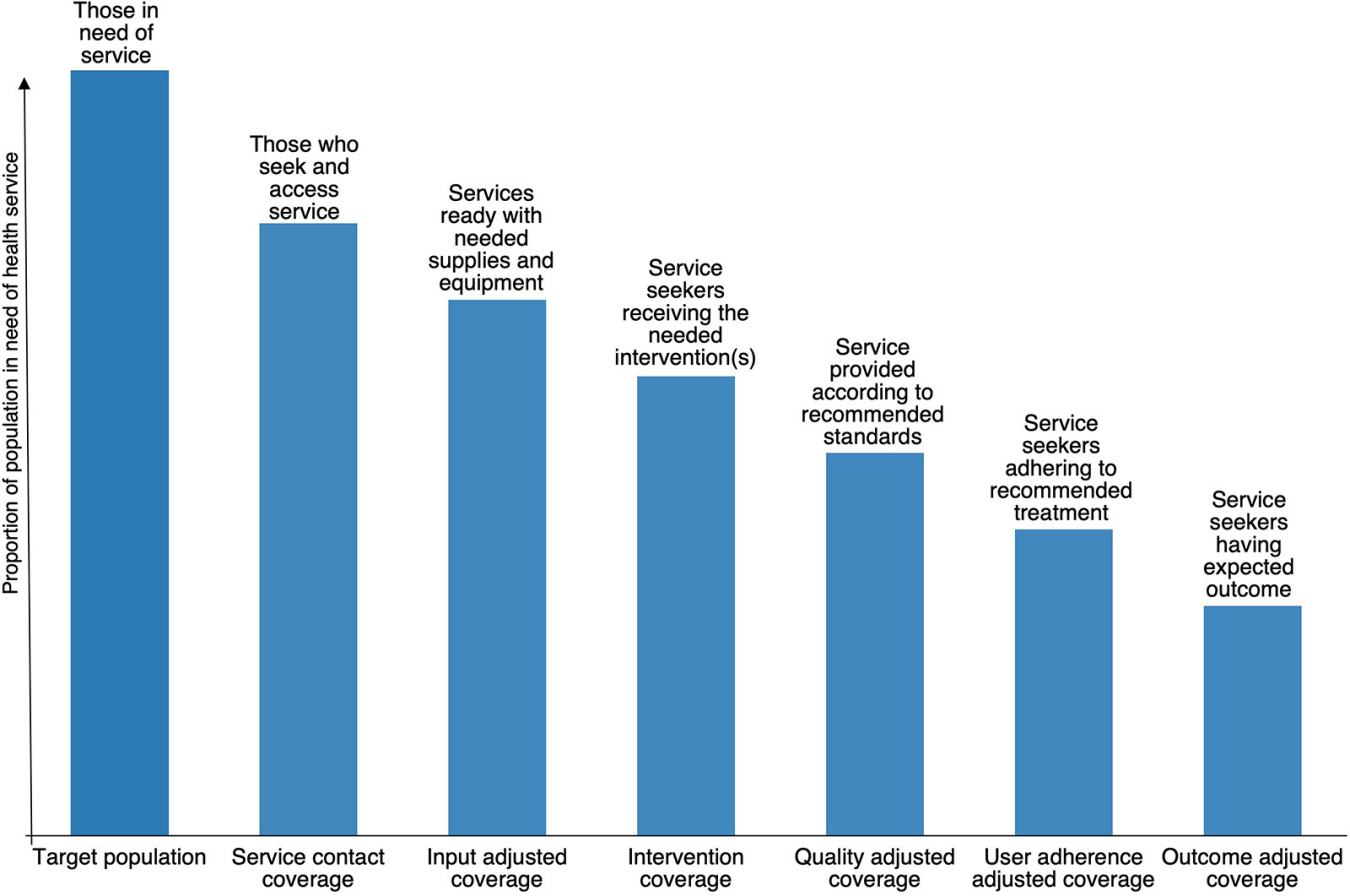
Cascade Describing the Seven Steps That the Target Population Moves Through From Service Contact Coverage to Outcome Adjusted Coverage^a^ Source: Adapted from Marsh et al.^6^ ^a^ Definitions for each stage of the cascade are provided at the top of each bar.

Ultimately, the Think Tank discussions facilitated consensus on how to define EC measurements in theory. However, due to the many feasibility challenges, data gaps, and limitations, there is yet to be a consensus on measuring EC in practice. A recent rapid systematic review on EC of lifesaving MNCH interventions and services in low- and middle-income countries found much heterogeneity in definitions and approaches toward measurement of EC when mapped against the EC cascade.^11^ There was particularly little consistency in the use of tracer items, composite, and summary measures for quality-adjusted coverage in the studies examined.^11^

### Using Effective Coverage Measures at the Global Level

Although the EC cascade provides a theoretical framework for measuring EC for an individual service or intervention, the measurement methods, including the selection of data for each step of the cascade, make using the cascade as a global MNCAH measurement tool challenging. We note that MNCAH EC measurement on a global scale is still premature and call for more research and validation across country contexts to reach universal consensus. We use examples from MNCAH services and current recommendations around quality of care (QOC) to highlight the complexities related to measuring EC for MNCAH, particularly for services that are intended to have multiple or poorly defined health outcomes.

## KEY CONSIDERATIONS RELATED TO MATERNAL, NEWBORN, CHILD, AND ADOLESCENT HEALTH

### Need for Maternal, Newborn, Child, and Adolescent Health Quality of Care Indicators

The Donabedian model proposes 3 categories of criteria for assessing QOC: inputs/structure, processes, and outcomes.^12^ The WHO’s “Standards for improving the quality of care” for MNCAH adapt this framework to provide guidance on how to deliver quality health services for MNCAH in health facilities.^12^^–^^15^ WHO has also used these standards as an organizing framework for developing QOC indicators. For example, in 2022, WHO published a set of 25 core indicators recommended for routine and periodic measurement of pediatric and young adolescent QOC in health facilities.^16^ However, by virtue of being core, these indicators were not developed to measure EC. Rather, they were developed to give high-level insights into the quality of services for children and young adolescents across the health system based on WHO QOC standards. Consequently, there is a lack of a parsimonious set of indicators that can reflect high-quality service delivery for individual MNCAH interventions. Indeed, care must be taken in selecting measurement proxies so as not to overemphasize the service delivery component at the expense of the QOC component. Furthermore, the choice of such proxies may be dependent on country setting, service type context, or availability of data.

### Accounting for the Complexity of Maternal, Newborn, Child and Adolescent Health Services and Interventions

The theoretical EC cascade, when taken at face value, assumes that a single intervention leads directly to a single measurable health outcome in a sequential fashion. In these instances, a measure of quality may be identified and successfully used in conjunction with service need and contact estimates to measure outcome-adjusted coverage. There are many examples of this type of use of health care cascades for specific conditions, such as TB treatment and measurement of EC for cataract surgery and refractive error correction.^17^^–^^20^ The communities of practice in these cases were able to align on measurement of the steps for their chosen cascades and on the outcome of interest. By contrast, many MNCAH interventions are provided as part of a continuum of essential services, each of which must be provided with quality for a positive health outcome. Measuring the EC of the service as a whole is a frequent request for MNCAH programs, a task made more difficult due to the complexity of health services, some of which do not have service quality measures that are feasible to collect and associated with positive health outcomes.

Measuring the EC of MNCAH services is made more difficult due to the complexity of health services, some of which do not have service quality measures that are feasible to collect and associated with positive health outcomes.

To illustrate the above challenge, take the example of a pregnant woman. She will need multiple antenatal care visits, with each visit covering several aspects of the pregnancy, all of which contribute to positive health outcomes for both the mother and the baby. The WHO guidelines for antenatal care currently include more than 20 different recommendations related to a wide range of aspects of care, including: nutritional interventions (e.g., micronutrient supplementation and dietary counseling); maternal interventions (e.g., screening for anemia, diabetes, HIV, syphilis, asymptomatic bacteriuria, and intimate partner violence, and preventive care, such as vaccinations); and fetal assessment (e.g., fetal growth measurements and morphology scans).^21^ The recommendations are also context-specific. For example, additional interventions are recommended for women living in malaria-endemic settings or in settings with low dietary calcium intake. Expected health outcomes from these interventions include positive pregnancy outcomes, but those outcomes are also heavily influenced by childbirth and postnatal care, as well as other factors, such as diet and environmental conditions. As a result, attributing health outcomes solely to antenatal care is challenging. As a result, MNCAH cascades are often estimated only through quality-adjusted coverage, which then serves as a proxy for EC.^11^

### Challenges With Data Sources and Data Quality

Once consensus is reached on what indicators to include for EC measurement, the next step is the challenge of capturing valid data for the target population, service contact, intervention coverage, and QOC measures. Comprehensive, high-quality data are essential for measuring and analyzing EC cascades and, in turn, determining the value and usefulness of EC measures. Lack of data availability means full EC cascades with recommended indicators may not be able to be constructed and measured. Poor data quality can result in under- or overestimations of EC, potentially leading to misdirected future quality planning and improvement activities. Improving the use of MNCAH indicators for global monitoring requires a comprehensive understanding of the global data landscape and how country-level data come together to provide global and national values for an indicator at a given point in time.^22^ Regardless of whether EC endpoints are measured directly through outcome-adjusted coverage or indirectly through quality-adjusted coverage, having valid data along the coverage cascade is essential for the results to be interpretable and useful for decision-making. In many cases, relying on a single data source to measure EC will not be adequate and, in some cases, not even possible. Instead, a combination and linkage of household survey data, health facility data, administrative data, or health management information systems is likely to be needed.^23^^–^^27^ However, current data gaps, especially in resource-limited settings, create considerable barriers to data access and data linkage for EC measurement. Data sources for quantifying the need for and use of many services or interventions have been identified and are in use by countries and the global public health community for monitoring programmatic success.^28^

Each source has strengths and limitations and captures different kinds of information, some representative of the national population, others specific to health facilities (i.e., health facility assessments or specific disease/condition registries). Population-based surveys, such as the Demographic Health Survey (DHS) or Multiple Indicator Cluster Survey, provide information on indicators describing certain target populations, health activities, and outcomes (e.g., health care seeking for acute illness or vaccination coverage) at the population level and can be combined with health facility assessments to provide data for many MNCAH quality-adjusted coverage estimates. However, the long lag time of household survey and health facility assessment reporting cycles means that available data reflect only past efforts. In contrast, routine health information systems (RHISs), such as the DHIS2, provide country-level data that are continuously available from service and/or individual record systems for program monitoring. By providing real-time data on the performance of health programs, RHISs have the potential to provide immediate, actionable data, currently missing from the above-mentioned surveys and be a major data source for EC measurement. However, RHISs are only representative of the services provided at health facilities and the individuals who can access these services, often omitting the most vulnerable populations. They also may not cover the entire health system with notable gaps, including the private sector and, in some cases, community providers, making the data useful mainly for improving the quality of the services reporting to the national public health system.^7^^,^^20^ Like RHISs, health facility assessments also do not cover community-based providers, who can be an important source of MNCAH services in some settings.

RHISs in resource-limited settings typically capture service utilization, intervention delivery, and outcomes but do not always capture service quality, limiting their use for measuring quality-adjusted coverage. A recent study measured EC of facility-based childbirth in Gombe State, Nigeria, and compared the capacity of 2 different data sources to measure EC.^29^ Based on the EC cascade, a list of indicators was selected to measure the first 4 steps of the cascade, with process quality-adjusted coverage acting as the proxy for EC. Authors linked population data from the Nigerian DHS with 2 different facility data sources: DHIS2 and primary health facility survey (HFS) data generated as part of a research project, as the HFS data were developed with QOC indicators as part of the research outcomes. When combining Nigerian DHS and HFS data, the authors were able to measure quality-adjusted coverage. However, when combining data from the Nigerian DHS with the DHIS2, measuring quality-adjusted coverage was not possible, as the data necessary for the selected indicators were not available. Although quality-adjusted coverage could potentially be tracked with data from the RHIS, this study demonstrates how data sources (in this case, DHS and DHIS2) that are typically available to decision-makers lack the needed information, in their current state, to measure EC.

Some of the challenges of using the health service coverage cascades to measure EC may be mitigated if the purpose of the cascade is identified up front. For example, different data sources will be used according to stakeholder needs and objectives. If the purpose is to monitor the health system’s overall performance, aggregate data from multiple facilities will be needed. By contrast, if the purpose is to measure a identify bottlenecks to achieving positive health outcomes at a health facility, then health facility records could be used. This distinction is crucial and can have policy implications for the health system being measured.

## RECOMMENDED ACTIONS TOWARD GAINING GLOBAL CONSENSUS ON EFFECTIVE COVERAGE MEASUREMENT

EC measurement is gaining momentum in the global public health community. Apart from the growing number of publications reporting on EC methodology and measurement in practice,^30^ several initiatives have been established to move the EC agenda forward. Since the late 2000s, the World Bank has invested more than US$2.5 billion in performance-based financing, presenting opportunities for advancing EC measurement.^31^ Additionally, in 2022, WHO established the Life Stage Quality of Care Metrics Technical Working Group (LSQM-TWG) for Maternal, Newborn, Child, and Adolescent Health and Ageing to support global and national QOC measurement efforts through developing and promoting harmonized methodologies, frameworks, guidance, and tools for QOC measurement across life stages, with EC viewed as a priority objective.^32^ These actions are needed to bring both clarity and a way forward to operationalize EC measurement at the country level. However, much work is still needed, and to take the next step, the LSQM-TWG participants endorsed the need for a call to action and a set of recommendations for improving data availability and quality and implementation research, as well as raising awareness of MNCAH EC measurement approaches and frameworks. The following recommendations build off the work of previous working groups and current evidence.^11^^,^^32^^–^^34^

EC measurement is gaining momentum in the global public health community.

### Dedicate Resources at Global and National Levels

Dedicate resources at global and national levels to improve data availability and quality for measuring quality-adjusted coverage of MNCAH services by:

- Investing in novel ways to collect intervention coverage and service quality data for facility-based and non-facility-based interventions, particularly in low- and middle-income countries
- Supporting facilities to collect measures of QOC, including patient experience, as part of routine care
- Dedicating resources toward technology and innovation to link data sources to support EC measurement analysis at the country level

### Prioritize Research Actions

Prioritize research actions to establish robust, comparable MNCAH EC measures by:

- Building consensus around measures of service readiness and quality across the MNCAH community of practice^35^^,^^36^
- Addressing how to link data sources better, in particular, linking RHISs with population-based data sources^30^
- Investing in research to address the challenges of operationalizing the EC cascade across different settings
- Investing in feasibility studies on valid tracer indicators of service readiness and quality to incorporate into all potential data sources, including RHISs

### Increase Awareness of Effective Coverage Frameworks

Increase awareness of MNCAH EC frameworks and sharing measurement approaches broadly by:

- Increasing dissemination of EC frameworks and QOC indicators over a range of platforms across intersectoral disciplines at global and national levels^6^^,^^7^^,^^13^^–^^15^
- Establishing an international community of practice to encourage the sharing of research findings and real-world EC measurement practices and facilitate robust debate about methodology and application

## CONCLUSIONS

Although standard MNCAH EC measures may be premature at the global level, increasing uptake and application of the EC cascade frameworks and measures within country service delivery planning and quality improvement activities will add real value to the growing body of knowledge and evidence toward harmonization of EC measurement practices and, in turn, perhaps lead to harmonized global level EC measurement in the future. Standardized EC measurement is a cornerstone for universal health coverage with quality services and is, ultimately, what we need in the SDG era to “leave no one behind.”

## Notes

**Peer Reviewed**

**First Published Online:** November 5, 2024.
